# Identification of Mango Cross-Reactive Allergens and Cross-Reactive Linear Epitopes Using Serum from Patients with Mango Allergy

**DOI:** 10.3390/ijms27114670

**Published:** 2026-05-22

**Authors:** Wenxuan Zhao, Honglei Guo, Yanjun Cong

**Affiliations:** Beijing Engineering and Technology Research Center of Food Additives, College of Food and Health, Beijing Technology and Business University, Beijing 100048, China; 1863443697@163.com (W.Z.); ghl1370780569@163.com (H.G.)

**Keywords:** mango, allergy, cross-reactive allergen, cross-reactive epitopes

## Abstract

Although mango is not classified among the nine major allergenic foods reported by the Food and Drug Administration (FDA), the increasing global and domestic consumption of mango has been accompanied by a growing number of reported cases of mango allergy. Currently, reports on cross-reactive allergens and cross-reactive linear epitopes in mango are limited. This study employed BLASTp (version 2.11.0+) to predict potential allergens that may cross-react with mango protein allergens and other food protein allergens. Subsequently, cross-reactive allergens were identified using sera from mango-allergic patients. Furthermore, similar sequences of the identified cross-reactive allergens were predicted by BLAST. These similar sequences were then synthesized by the solid-phase peptide synthesis method. Finally, the cross-reactive linear epitopes were determined by assessing their IgE-binding capacity using serum IgE from the same patient cohort. The results demonstrated that the sera from mango-allergic patients exhibited IgE-binding cross-reactivity with those from peanut, wheat, cashew, pistachio, and hazelnut, particularly with IgE-binding cross-reactivity to wheat and hazelnut, which has not been previously reported. The following novel cross-reactive linear epitopes were identified: the AA80–88 sequence of mango chitinase with the AA37–45 sequence of wheat Tri a 27 and the AA15–22 sequence of mango profilin with the AA65–72 sequence of pistachio Pis v 1. Furthermore, multiple cross-reactive epitopes were mapped between mango profilin and peanut Ara h 5, corresponding to the sequences AA31–51/AA31–50, AA50–65/AA52–65, AA76–96/AA76–96, and AA103–117/AA104–117, respectively.

## 1. Introduction

Mango is a widely consumed tropical fruit worldwide. With its rising global consumption, there has been a corresponding increase in reports of mango allergy [1,2,3,4]. The FDA reported nine major categories of allergenic foods, which primarily include high-protein foods such as milk and eggs [5]. Although mango, as a fruit, is not listed among them, numerous studies reporting its high allergenic prevalence necessitate increased attention [6]. Six major allergenic proteins in mango have been identified based on the literature and the Allergome database (https://allergen.org/, accessed on 21 September 2021), including class IV chitinase (Man i 1), PR-10 protein/Bet v1-related protein (Man i 2), profilin (Man i 4), class IV chitinase, β-1,3-glucanase, lipoxygenase, and Bet v 1-like homologous protein (Man i 14 kDa) [7,8,9]. In addition to the officially designated allergen, other proteins such as glyceraldehyde-3-phosphate dehydrogenase (GAPDH) have also been reported to bind IgE from mango-allergic patients and are considered potential allergens. Research on mango epitopes is still limited. While bioinformatic predictions have been conducted for mango allergens, these allergens have yet to be substantiated by robust experimental validation, such as serological testing or animal models. Food IgE-binding cross-reactivity refers to the phenomenon where IgE antibodies or T cells specific to one allergen can recognize and react to similar or structurally unrelated allergens from other sources, often through shared B-cell or T-cell epitopes [10]. Currently, studies on mango cross-reactive allergens rely mainly on bioinformatic predictions, which can identify potential epitope similarities with known allergens from related species or protein families. Nevertheless, the precise mechanisms governing IgE-binding cross-reactivity among different foods have not yet been fully elucidated. Consequently, with the increasing recognition of the importance of allergenic IgE-binding cross-reactivity, research into its mechanisms has become imperative. Studies on mango IgE-binding cross-reactivity will establish a theoretical foundation for guiding dietary choices among mango-allergic individuals, preventing cross-reactive allergies, and ultimately contributing to public health assurance.

This study utilized BLASTp to predict potential allergens that may cross-react with mango protein allergens and other food protein allergens. Subsequently, cross-reactive allergens were identified serologically using IgE from mango-allergic patients as a probe, and Western blotting was subsequently employed to detect food proteins that interacted with the patients’ serum antibodies. Furthermore, similar sequences of the identified cross-reactive allergens were predicted using BLASTp. These sequences were then synthesized via the solid-phase peptide synthesis method. Finally, the cross-reactive linear epitopes were identified by assessing their IgE-binding capacity with patient serum.

## 2. Results

### 2.1. Protein Concentration Results of Mango and Other Foods

The protein concentrations of mango, wheat, peanut, shrimp, almond, hazelnut, pistachio, cashew, carrot, peach, lychee, crystal pear, fragrant pear, apple, and banana were determined using the Coomassie Brilliant Blue method [11], and the results are shown in Table S1. Among these, the hazelnut extract presented the highest protein concentration at 37.372 mg/mL, followed by peanut (17.607 mg/mL), shrimp (15.059 mg/mL), almond (12.247 mg/mL), pistachio (7.875 mg/mL), and cashew (5.498 mg/mL). Wheat protein concentration was 1.495 mg/mL. These protein concentrations were relatively high compared to those of fruits and vegetables, where crystal pear contained 0.415 mg/mL, mango 0.389 mg/mL, fragrant pear 0.251 mg/mL, lychee 0.180 mg/mL, banana 0.088 mg/mL, apple 0.072 mg/mL, peach 0.065 mg/mL, and carrot 0.056 mg/mL. Figure S1 shows the standard curve for protein concentration determination by the Coomassie Brilliant Blue method, with R^2^ > 0.999.

### 2.2. SDS-PAGE Analysis of Mango and Other Food Proteins

#### 2.2.1. SDS-PAGE Analysis of Mango Proteins

**Figure 1:**
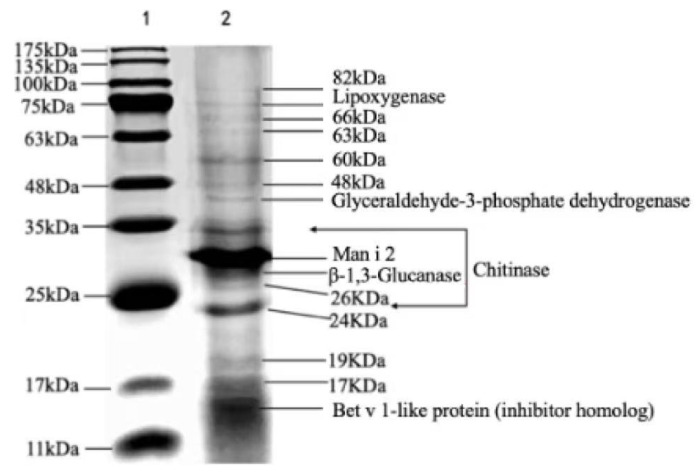
SDS-PAGE electrophoresis profiles. Lane 1 was loaded with the protein molecular weight marker, and lane 2 contained the mango protein extract.

The SDS-PAGE profile of mango proteins is shown in Figure 1. Lane 1 corresponds to the protein molecular weight marker. Lane 2 contains the mango protein extract, displaying bands from top to bottom at approximately 82, 75 (lipoxygenase), 66, 63, 60, 48, 40 (glyceraldehyde-3-phosphate dehydrogenase), 35, 34, 30 (Man i 2), 27 (β-1,3-glucanase), 26, 24, 19, 17, and 14 kDa (inhibitor/Bet v 1-like homologous protein).

#### 2.2.2. SDS-PAGE Profiles of Hazelnut, Cashew, Pistachio, Wheat, Peanut, Shrimp and Almond

**Figure 2:**
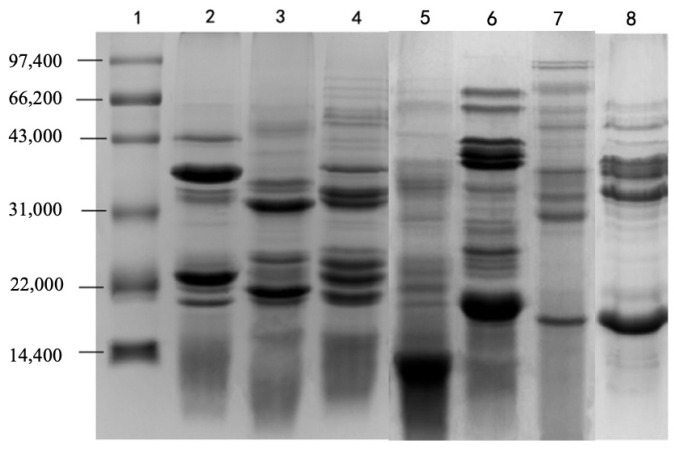
SDS-PAGE profiles. Lane 1: protein molecular weight marker; lane 2: hazelnut; lane 3: cashew; lane 4: pistachio; lane 5: wheat; lane 6: peanut; lane 7: shrimp; lane 8: almond.

SDS-PAGE analysis was performed for hazelnut, cashew, and pistachio, with the results presented in Figure 2. Lane 1 was loaded with the protein molecular weight marker. In lane 2 (hazelnut), bands were observed from top to bottom at approximately 72, 62, 43, 42, 37, 35 (Cor a 6), 33, 29, 24, 23, 21, 20, 17 (*Cor a 1/Cor a 12*), and 14 kDa (Cor a 2). Lane 3 (cashew) displayed bands at approximately 60, 55 (Ana o 2), 50 (Ana o 1), 38, 34, 33, 32, 30, 28, 25, 24, 22, 20, 17, and 14 kDa (Ana o 3). In lane 4 (pistachio), bands were detected at approximately 81, 74, 67, 58, 55 (Pis v 3), 51, 43, 41, 36 (Pis v 5), 34, 32 (Pis v 2), 30, 29, 26 (Pis v 4), 24, 23, 20, 17, and 14 kDa. In lane 5 (wheat), bands were detected from top to bottom at approximately 75, 65 (Tri a 19), 61, 43, 40 (Tri a 36), 37, 36, 35 (Tri a 20), 32, 31, 29, 27 (Tri a 27), 25, 24, 22, 18, and 16 kDa (Tri a 30). Lane 6 (peanut) displayed bands at approximately 64 (Ara h 1), 60 (Ara h 3), 43, 40, 38, 35, 34, 33, 31, 30, 28, 27, 26, 25, 22, 20, 17 (*Ara h 2/Ara h 8/Ara h 15*), and 15 kDa (*Ara h 5/Ara h 6/Ara h 7*). In lane7 (shrimp), bands were observed at approximately 87, 83, 69, 56, 45 (Cra c 2), 40 (Pen m 2), 38 (*Cra c 1/Pen m 1*), 36 (*Lit v 1/Pen a 1*), 32, 30, and 21 kDa (Cra c 6). In lane 8 (almond), bands were observed from top to bottom at approximately 66, 63, 60 (Pru du 10), 45 (Pru du γ), 42, 41, 40, 37, 36 (Pru du 6), 34, 32, 31 (Pru du 8), 28, 23 (Pru du 2), 22 (Pru du 6), 18, 17 (Pru du 1), and 14 kDa (Pru du 4).

#### 2.2.3. SDS-PAGE Profiles of Carrot, Peach, Litchi, Crystal Pear, Fragrant Pear, Banana, and Apple

**Figure 3:**
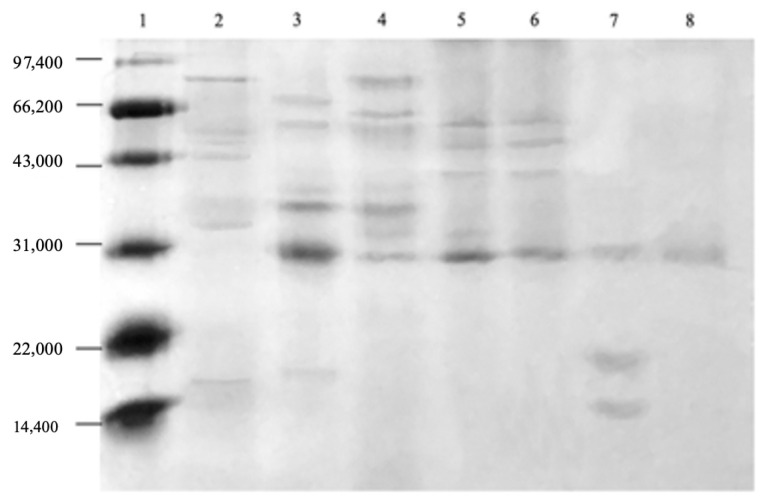
SDS-PAGE profiles. Lane 1: protein molecular weight marker; lane 2: carrot; lane 3: peach; lane 4: litchi; lane 5: crystal pear; lane 6: fragrant pear; lane 7: banana; lane 8: apple.

The SDS-PAGE profiles of carrot, peach, litchi, crystal pear, fragrant pear, banana, and apple are shown in Figure 3. Lane 1 was loaded with the protein molecular weight marker. In lane 2 (carrot), bands were observed from top to bottom at approximately 89, 50, 48, 43, 34, and 16 kDa (Dau c 1, pathogenesis-related protein). Lane 3 (peach) displayed bands at approximately 68, 60, 39, 37, 34, 31, and 17 kDa. Bands in lane 4 (litchi) were detected at approximately 90, 66, 60, 39, 37, 34, and 31 kDa. In lane 5 (crystal pear), bands were present at approximately 60, 45, 40, 34 (Pyr p 5), and 31 kDa. Lane 6 (fragrant pear) showed bands at approximately 60, 45, 40, and 31 kDa. In lane 7 (banana), bands corresponded to approximately 30 (Mus a 5), 20 (Mus a 4), and 15 kDa (Mus a 1). A band at approximately 31 kDa was observed in lane 8 (apple).

### 2.3. Prediction Results of Mango Cross-Reactive Allergens

According to the Codex Alimentarius Commission (CAC), potential cross-sensitization should be considered—indicating the presence of potential IgE-binding cross-reactivity—if any sequence exhibits greater than 35% similarity over 80 amino acids or contains eight consecutive identical amino acids [12]. To increase the accuracy of subsequent serological validation, sequences whose similarity exceeded 30% were also classified as potentially cross-reactive allergens. A limitation of our in silico approach is the use of a relaxed sequence identity threshold (≥30%) for initial screening, which increases sensitivity but may also include false positives. This choice was made to maximize the discovery of candidate cross-reactive allergens for empirical testing, and all reported conclusions are supported by subsequent serological IgE-binding data. The prediction results of mango allergens cross-reactive via BLAST are presented in Table 1. As shown in Table 1, mango profilin (Man i 4) exhibited consistently high sequence identity (>75%) with profilins from multiple plant sources (e.g., peanut Ara h 5, almond Pru du 4, hazelnut Cor a 2, peach Pru p 4, lychee Lit c 1, apple Mal d 4, banana Mus a 1, pear Pyr c 4, and carrot Dau c 4), suggesting that profilin may be a major driver of IgE-binding cross-reactivity. In contrast, other mango allergens (e.g., chitinase and β-1,3-glucanase) showed more limited or moderate similarity to selected food allergens. Mango glyceraldehyde-3-phosphate dehydrogenase has >30% similarity with wheat Tri a 34. The mango chitinase presented >30% similarity with those of wheat Tri a 37, hazelnut Cor a 1, Cor a 12, and banana Mus a 2. Mango profilin exhibited >30% similarity with wheat Tri a 12, Tri a 29, Tri a 30, Tri a 37; peanut Ara h 5; almond Pru du 4; shrimp Cra c 6; hazelnut Cor a 2 and Cor a 13; pistachio Pis v 1; peach Pru p 4; lychee Lit c 1; banana Mus a 1; apple Mal d 4; pear Pyr c 4; carrot Dau c 4; and banana Mus a 5. Compared with banana Mus a 5, mango β–1,3-glucanase shares >30% similarity.

### 2.4. Immunoblot Results of Mango Cross-Reactive Allergens Identified by Sera from Allergic Patients

#### 2.4.1. Immunoblot Analysis of Mango, Wheat, Peanut, Cashew, Pistachio, Hazelnut and Almond Allergens Recognized by Sera from Mango-Allergic Patients

**Figure 4:**
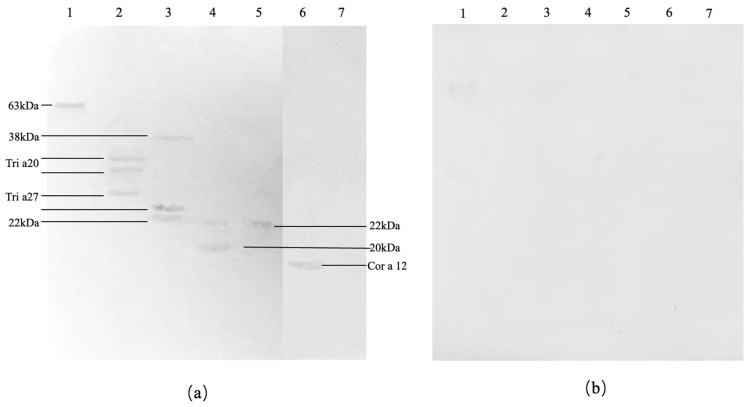
Western blot of mango, wheat, peanut, cashew, pistachio, hazelnut and almond with mango-allergic patients’ serum antibody. (**a**) Immunoblot with positive serum; (**b**) Immunoblot with negative control serum; 1: mango, 2: wheat, 3: peanut, 4: cashew, 5: pistachio, 6: hazelnut, 7: almond. Protein loads were not normalized across lanes (see Table S1 for individual extract concentrations); therefore, band intensities should not be directly compared between different food extracts. The figure is intended for qualitative detection of reactive bands.

Mango, wheat, peanut, cashew, pistachio, hazelnut and almond react with antibodies in the serum of mango-allergic patients, as shown in Figure 4a. One band at about 63 kDa was detected in mango. To exclude false-positive reactions, immunoblotting was performed using negative control sera from healthy individuals, as presented in Figure 4b. No reaction was observed between mango and the negative control sera. Therefore, in the immunoblot, a dominant IgE-reactive band was observed at approximately 63 kDa. No other bands were reliably detected under our experimental conditions, possibly due to lower protein abundance or suboptimal transfer of smaller proteins (e.g., chitinase or profilin). In the wheat extract, three IgE-reactive bands were observed at approximately 35 kDa, 31 kDa, and 27 kDa. Their migration positions are suggestive of the known wheat allergens Tri a 20 (~35 kDa) and Tri a 27 (~27 kDa), as well as an additional putative allergen at 31 kDa. Peanut presented three bands at 38 kDa, 25 kDa, and 22 kDa. To exclude false-positive reactions, immunoblot analysis was performed using serum from healthy individuals as a negative control, as presented in Figure 4b. Neither wheat nor peanut reacted with the negative control serum. Therefore, wheat specifically reacts with sera from mango-allergic patients, resulting in three cross-reactive allergen bands: Tri a 20 kDa (35 kDa), 31 kDa, and Tri a 27 kDa (27 kDa). Similarly, peanut specifically reacted with the sera from mango-allergic patients, resulting in three cross-reactive allergen bands at 38 kDa, 25 kDa, and 22 kDa. Song et al. [8] previously reported high sequence similarity between mango profilin and peanut Ara h 5 profilin, although this was not experimentally validated. Cashew displayed two bands at approximately 22 kDa and 20 kDa, while pistachio showed one band at 22 kDa. To exclude false-positive reactions, immunoblot analysis was performed using serum from healthy individuals as a negative control, as presented in Figure 4b. Neither cashew nor pistachio reacted with the negative control serum. Therefore, it can be concluded that cashew specifically reacted with sera from mango-allergic patients, resulting in two cross-reactive allergen bands at 22 kDa and 20 kDa, whereas pistachios presented one cross-reactive allergen band at 22 kDa. Bastiaan-Net et al. [13] reported substantial IgE-binding cross-reactivity between mango and cashew via inhibitory immunoblotting using sera from cashew-allergic patients, suggesting that chitinase and β-1,3-glucanase may be involved in this IgE-binding cross-reactivity. No specific IgE binding was observed for the almond extract, whereas the hazelnut extract displayed immunoreactivity with serum from mango-allergic patients. A single IgE-reactive band was detected in hazelnut at approximately 17 kDa, a migration position suggestive of the known hazelnut allergen Cor a 12. To exclude false-positive reactions, immunoblot analysis was performed using serum from healthy individuals as a negative control, as presented in Figure 4b. Neither the hazelnut nor the almond reacted with the negative control serum. Therefore, it can be concluded that hazelnuts specifically react with sera from mango-allergic patients, resulting in one cross-reactive allergen band, Cor a 12 (17 kDa), indicating the presence of a cross-reactive allergen between hazelnuts and mango.

#### 2.4.2. Immunoblot Analysis of Lychee, Banana, Apple, Carrot, Fragrant Pear, Crystal Pear, Peach and Shrimp Allergens Recognized by Sera from Mango-Allergic Patients

**Figure 5:**
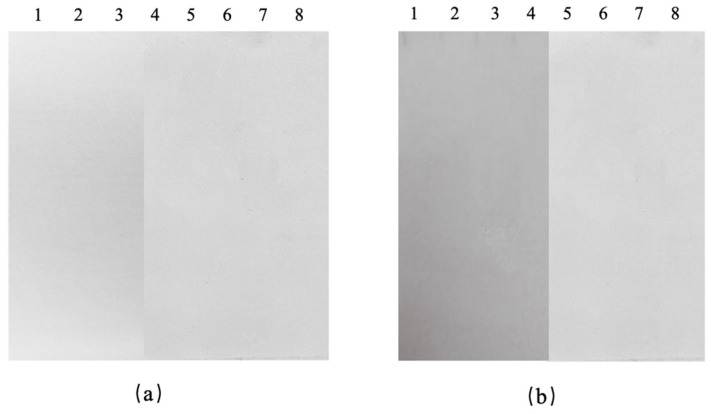
Western blot of litchi, bananas, apples, carrots, pears, crystal pears, peaches, and shrimps with mango-allergic patients’ serum antibody. (**a**) Immunoblot with positive serum; (**b**) Immunoblot with negative control serum; 1: lychee, 2: banana, 3: apple, 4: carrot, 5: fragrant pear, 6: crystal pear, 7: peach, 8: shrimp.

Lychee, banana, and apple samples exhibited no immunoreactivity to either serum antibodies from mango-allergic patients or negative control sera, as shown in Figure 5. Song et al. [8] reported high sequence similarity between mango profilin and apple Mal d 4 profilin, although this was not experimentally validated. Yan et al. [14] demonstrated high homology between mango and lychee profilins through amino acid sequence alignment, suggesting a potential basis for IgE-binding cross-reactivity, but no experimental confirmation was performed. Carrot, fragrant pear, crystal pear, peach, and shrimp showed no immunoreactivity with serum antibodies from either mango-allergic patients or negative controls, as shown in Figure 5. Song et al. [8] predicted high sequence similarity between mango profilin and profilins from pear (Pyr c 4), peach (Pru p 4), apple (Mal d 4), peanut (Ara h 5), and carrot (Dau c 4), though these predictions lack experimental validation. In each immunoblot experiment, the mango protein extract served as an internal positive control. The consistent detection of the 63 kDa IgE-reactive band in the mango lane across all experiments (Figure 4 and Figure 5) confirmed that the serum application, secondary antibody, and detection system were functioning properly in every assay. Therefore, the absence of reactive bands in other food extracts on the same membranes can be attributed to genuine lack of cross-reactivity rather than experimental failure.

### 2.5. Prediction Results of Similar Sequences of Mango Cross-Reactive Allergens

The similar sequences between mango and its cross-reactive allergens (peanut, wheat, pistachio, hazelnut, and cashew) were predicted, and the results are presented in Table 2, Table 3 and Table 4. The predicted potential functional epitopes include: mango chitinase with wheat Tri a 27; mango profilin (Man i 4) with peanut Ara h 5; and mango profilin with pistachio Pis v 1.

### 2.6. Identification of Cross-Reactive Linear Epitopes in Mango and Cross-Reactive Allergens by Sera from Mango-Allergic Patients

**Figure 6:**
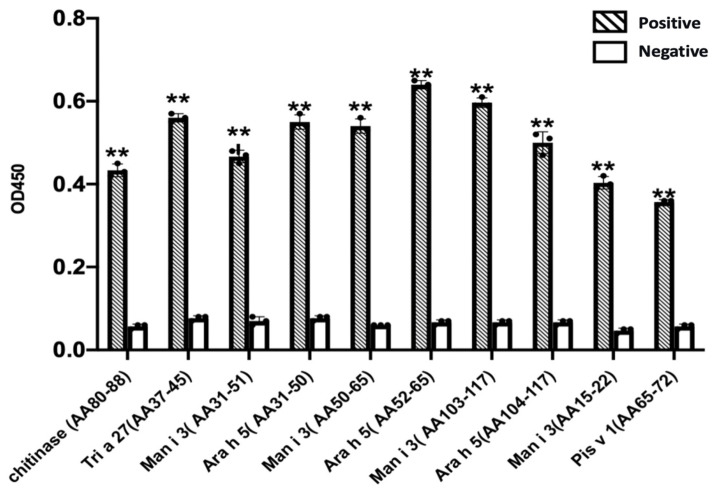
Results of serum recognition epitopes in mango allergy patients. Note: Chitinase AA80–88 represents the AA80–88 sequence of mango chitinase; Tri 27 AA37–45 represents the AA37–45 sequence of wheat Tri a 27; Man i 4 AA31–51 represents the AA31–51 sequence of mango profilin; Ara h 5 AA31–50 represents the AA31–50 sequence of peanut Ara h 5; Man i 4 AA50–65 represents the AA50–65 sequence of mango profilin; Ara h 5 AA52–65 represents the AA52–65 sequence of peanut Ara h 5; Man i 4 AA103–117 represents the AA103–117 sequence of mango profilin; Ara h 5 AA104–117 represents the AA104–117 sequence of peanut Ara h 5; Man i 4 AA15–22 represents the AA15–22 sequence of mango profilin; Pis v 1 AA65–72 represents the AA65–72 sequence of pistachio Pis v 1.

Following the identification of cross-reactive food proteins by immunoblotting, peptide-based ELISA was employed to map and confirm the specific linear IgE-binding epitopes within those proteins; the statistical analysis of these ELISA results using serum IgE from mango-allergic patients is presented in Figure 6. The absorbance values of the similar sequences in the sera from mango-allergic patients were significantly greater than those in the negative control sera (** *p* < 0.05), indicating that all the predicted similar sequences are cross-reactive linear epitopes. The specific results are as follows: the AA80–88 sequence of mango chitinase and the AA37–45 sequence of wheat Tri a 27 constitute a cross-reactive epitope; the AA15–22 sequence of mango profilin and the AA65–72 sequence of pistachio Pis v 1 constitute a cross-reactive epitope; the AA31–51 sequence of mango profilin and the AA31–50 sequence of peanut Ara h 5 constitute a cross-reactive epitope; the AA50–65 sequence of mango profilin and the AA52–65 sequence of peanut Ara h 5 constitute a cross-reactive epitope; the AA76–96 sequence of mango profilin and the AA76–96 sequence of peanut Ara h 5 constitute a cross-reactive epitope; and the AA103–117 sequence of mango profilin and the AA104–117 sequence of peanut Ara h 5 constitute a cross-reactive epitope. It should be noted that while immunoblotting revealed IgE-reactive bands for hazelnut (Cor a 12) and cashew, these were not included in the subsequent peptide-based ELISA analysis shown in Figure 6. For cashew, BLAST analysis did not predict any significant sequence similarity with mango allergens (Table 1), and therefore no candidate peptides could be designed for testing. For hazelnut, although sequence similarity was predicted between mango chitinase and Cor a 12, the corresponding peptide was not included in the initial synthesis due to resource limitations and the priority given to epitopes with higher sequence identities. Future studies should include these peptides to comprehensively map all potential cross-reactive epitopes identified by immunoblotting.

Following the identification of cross-reactive food proteins by immunoblotting, peptide-based ELISA was employed to precisely map and confirm the specific linear IgE-binding epitopes within those proteins.

Tsai et al. [15] analyzed the B-cell epitopes of mango glyceraldehyde-3-phosphate dehydrogenase and suggested that the sequences 154–164, 215–222, and 318–326 are potential B-cell epitopes. Yan et al. [14] analyzed the structural domains of mango profilin and chitinase via SMART software v10 (https://smart.embl.de) and proposed that the structural domains may represent functional epitopes of profilin or chitinase. Cao et al. [16] validated these findings through serological experiments and identified the 8–28 aa sequence (chitin-binding domain) as the major linear IgE-binding epitope of chitinase. Zhang et al. [17] analyzed the B-cell epitopes of mango chitinase and identified potential regions as follows: 38–46, 64, 67, 75–76, 89, 97–101, 115, 122, 124–126, 128–129, 133–138, 144–150, 152, 176, 178–181, and 222–225.

## 3. Discussion

Numerous studies have reported on the sensitization characteristics of individual mango allergens, whereas research on mango cross-reactive allergens remains relatively limited. This area represents a challenging and active research focus, with current investigations relying primarily on bioinformatic predictions. Paschke et al. [7] identified the 14 kDa protein in mango that exhibited IgE-binding cross-reactivity with birch pollen Bet v 1 through inhibitory immunoblotting. Furthermore, using sera from mango-allergic patients, they demonstrated that proteins of about 40, 43, and 67 kDa in mango were primarily responsible for IgE-binding cross-reactivity with carrot. Song et al. [8] predicted high sequence similarity between mango profilin and profilins from pear (Pyr c 4), peach (Pru p 4), apple (Mal d 4), peanut (Ara h 5), and carrot (Dau c 4), although experimental validation was not conducted. Yan et al. [14] performed homology alignment of profilin amino acid sequences from various fruits and revealed high homology among mango, grape, peach, citrus, and lychee, suggesting a potential basis for IgE-binding cross-reactivity, yet experimental confirmation has not been performed. Funes et al. [18] demonstrated IgE-binding cross-reactivity among pistachio, cashew, and mango within the Anacardiaceae family using RAST inhibition. In 2018, Cardona et al. [9] used immunoblotting with sera from mango-allergic patients and suggested potential IgE-binding cross-reactivity between mango and three banana components: Mus a 1, Mus a 2, and Mus a 5. Similarly, Bastiaan-Net et al. [13] employed immunoblotting with cashew-allergic sera and revealed substantial IgE-binding cross-reactivity between mango and cashew, potentially associated with chitinase and β-1,3-glucanase.

In this study, using serum IgE from mango-allergic patients as a probe, we systematically and scientifically verified mango cross-reactive allergens through immunoblotting. Sera from mango-allergic patients exhibited IgE-binding cross-reactivity with those from wheat, peanut, cashew, pistachio, and hazelnut. This study innovatively identified wheat and hazelnuts as cross-reactive allergens of mango. Furthermore, previously unreported cross-reactive allergen bands were detected by immunoblotting. The proteins that showed IgE-binding cross-reactivity with serum antibodies from mango-allergic patients included the following: wheat proteins with molecular weights of Tri a 20 (35 kDa), 31 kDa, and Tri a 27 (27 kDa); peanut proteins of 38 kDa, 25 kDa, and 22 kDa; cashew proteins of 22 kDa and 20 kDa; pistachio protein of 22 kDa; and hazelnut protein Cor a 12 (17 kDa). For future research, isolation and purification of individual mango allergens and preparation of monoclonal antibodies to further characterize mango cross-reactive allergens are recommended.

Currently, reports on the B-cell epitopes of individual mango allergens are limited, and no studies have reported cross-reactive linear epitopes. Yan et al. [14] analyzed the structural domains of mango profilin and chitinase using SMART software and proposed that these domains may represent linear epitopes of profilin or chitinase. Tsai et al. [15] analyzed B-cell epitopes of mango glyceraldehyde-3-phosphate dehydrogenase and suggested that the sequences 154–164, 215–222, and 318–326 may serve as potential B-cell epitopes. Cao et al. [16] experimentally confirmed the aa8–28 sequence as the major linear IgE-binding epitope of chitinase. Zhang et al. [17] analyzed B-cell epitopes of mango chitinase and identified potential regions as follows: 38–46, 64, 67, 75–76, 89, 97–101, 115, 122, 124–126, 128–129, 133–138, 144–150, 152, 176, 178–181, and 222–225. This study, for the first time, identified cross-reactive linear epitopes of mango: the AA80–88 sequence of mango chitinase with the AA37–45 sequence of wheat Tri a 27; the AA15–22 sequence of mango profilin with the AA65–72 sequence of pistachio Pis v 1; the AA31–51 sequence of mango profilin with the AA31–50 sequence of peanut Ara h 5; the AA50–65 sequence of mango profilin with the AA52–65 sequence of peanut Ara h 5; the AA76–96 sequence of mango profilin with the AA76–96 sequence of peanut Ara h 5; and the AA103–117 sequence of mango profilin with the AA104–117 sequence of peanut Ara h 5. Although bioinformatic analysis predicted potential cross-reactivity between mango allergens and several other foods (e.g., almond, apple, peach; Table 1), immunoblotting failed to detect IgE binding for these foods. Several factors may account for this discrepancy. First, the predicted linear epitopes might be conformationally masked in the native three-dimensional structure, rendering them inaccessible to IgE antibodies even if the linear sequence is present. Second, post-translational modifications (e.g., glycosylation) may differ between mango and these foods, altering epitope recognition. Third, the abundance or integrity of the putative cross-reactive protein in the extract might be low due to degradation or incomplete extraction, falling below the detection threshold of immunoblotting. Fourth, although the sequence identity exceeded the 30% threshold, the critical amino acid residues required for IgE binding may not be sufficiently conserved. These observations underscore that bioinformatic similarity alone is not sufficient to predict IgE-binding cross-reactivity, and experimental validation remains essential.

One might question why the immunoblot of mango extract showed only a prominent 63 kDa IgE-reactive band, whereas the peptide ELISA demonstrated strong IgE binding to synthetic peptides derived from mango chitinase (~30 kDa) and profilin (~14 kDa). This apparent discrepancy can be explained by several technical factors. First, peptide ELISA is intrinsically more sensitive than immunoblotting, especially for detecting low-abundance IgE specific for linear epitopes. Second, small proteins such as profilin often transfer poorly onto PVDF membranes during electroblotting, particularly under standard conditions optimized for larger proteins, leading to weak or absent signals. Third, the crude mango extract contains a highly abundant 63 kDa protein that may dominate the immunoblot signal and obscure weaker bands. Thus, the absence of chitinase and profilin bands on the immunoblot does not invalidate the ELISA results; rather, it reflects the lower detection sensitivity of immunoblotting for these particular proteins. The ELISA findings are robust (positive and negative controls performed as expected) and constitute the primary evidence for the identified cross-reactive linear epitopes. The immunoblot of mango extract showed only a dominant 63 kDa IgE-reactive band, with no detectable bands at the expected molecular weights of well-known mango allergens such as chitinase (~30 kDa) or profilin (~14 kDa). Several factors explain this. First, technical limitations: small proteins often transfer poorly onto PVDF membranes, and the crude extract may have partially degraded labile allergens while the 63 kDa protein remained intact. Second, our patient cohort (from Zhejiang, China) may have an atypical sensitization profile, mounting a dominant IgE response against a higher-molecular-weight allergen rather than typical profilins or PR-10 proteins. The 63 kDa band likely corresponds to a known allergen such as glyceraldehyde-3-phosphate dehydrogenase (possible dimer or post-translationally modified form) or an uncharacterized glycoprotein; definitive identification requires mass spectrometry. Importantly, the absence of low-molecular-weight bands does not contradict the strong peptide ELISA results for chitinase and profilin, because ELISA uses purified synthetic peptides at high density, bypassing transfer and abundance issues. Thus, the two methods are complementary. A limitation of the immunoblot analysis is that equal volumes rather than equal protein masses were loaded, resulting in variable protein amounts across lanes (Table S1). Therefore, band intensities were not compared quantitatively between different food extracts; only qualitative detection (presence or absence of bands) is reported. This does not affect the identification of novel cross-reactive foods or the peptide ELISA results.

Owing to the limitations of bioinformatics, the prediction of cross-reactive allergens has inherent uncertainty. In vitro serological identification using IgE antibodies is a commonly employed method to validate these predictions. As polyclonal IgE antibody reactions often yield false positives, the inclusion of negative serum controls is crucial to ensure the accuracy of the results. Currently, the IgE antibodies used in experiments are either prepared based on allergen-specific epitopes or epitopes capable of inducing IgE-binding cross-reactivity. The proportion of cross-reactive IgE produced depends on the patient’s genetic factors [19]. Some studies suggest that immunological IgE-binding cross-reactivity is specific and occurs primarily when serum IgE antibodies from allergic patients bind to conserved epitopes of allergens. The strength of IgE-binding cross-reactivity is associated with the affinity of IgE antibodies; however, not all IgE cross-reactions translate to clinical IgE-binding cross-reactivity, making in vivo validation essential. The mango cross-reactive allergens and cross-reactive linear epitopes identified in this study by immunoblotting should be further validated through future animal experiments. In the context of food allergy, linear IgE-binding epitopes appear to be more relevant since conformational epitopes are easily degraded by gastrointestinal digestion [20]. Therefore, this study focused primarily on identifying mango cross-reactive linear epitopes. We acknowledge that the relatively small sample size (n = 5) is a limitation of this exploratory study. While this cohort size is appropriate for initial allergen and epitope discovery, larger patient populations will be necessary to validate the generalizability of these findings and to assess the clinical relevance of the identified cross-reactive epitopes through quantitative inhibition assays and oral food challenges. In summary, while 5 patients is not sufficient for prevalence estimates, it is appropriate for the exploratory, mechanism-focused aims of this study.

The identification of wheat and hazelnut as novel cross-reactive allergens of mango suggests that mango-allergic individuals may be at risk of unexpected reactions to wheat- or hazelnut-containing foods. Clinicians may consider screening for IgE reactivity to these foods in patients with unexplained post-ingestion symptoms.

Mechanistically, the multiple cross-reactive epitopes mapped between mango profilin (Man i 4) and peanut Ara h 5 support profilin as a pan-allergen driving mango–peanut cross-reactivity. Moreover, the shared short peptide motif between mango chitinase and wheat Tri a 27 (RDGFLNAAN vs. RDGLLDAAN) raises the hypothesis that even evolutionarily distant species can confer IgE-binding cross-reactivity through conserved short linear epitopes, independent of full-length protein homology. These hypotheses warrant validation by inhibition assays and clinical studies.

In addition to the sample size, the use of a serum pool from the five patients, while practical for initial screening, has inherent limitations. Pooled sera do not capture inter-individual variability in IgE reactivity; it remains possible that some of the observed cross-reactive bands or epitopes are driven by one or two highly sensitized individuals rather than being representative of the entire cohort. Whenever serum volume permits, individual serum analysis is preferred to demonstrate the consistency of cross-reactive patterns. Due to the limited volume of serum available for this exploratory study, such individual analyses could not be performed. Therefore, our findings should be considered hypothesis-generating, and future studies are required to validate the identified cross-reactive allergens and linear epitopes using individual sera from larger patient cohorts, ideally combined with quantitative inhibition assays.

## 4. Materials and Methods

### 4.1. Materials and Reagents

Bovine serum albumin (BSA), 4-chloro-1-naphthol, acrylamide, N,N′-methylenebisacrylamide, β-mercaptoethanol, Tris base, glycine, sodium dodecyl sulfate (SDS), dimethyl sulfoxide (DMSO), Tween-20, and 3,3′,5,5′-tetramethylbenzidine (TMB) were purchased from Sigma-Aldrich (St. Louis, MO, USA). Coomassie Brilliant Blue R-250 was obtained from Beijing Banxia Science & Technology Development Co., Ltd. (Beijing, China). The low-molecular-weight protein standard was procured from TIANGEN Biotech (Beijing, China). Polyvinylidene fluoride (PVDF) membranes were obtained from Millipore (Billerica, MA, USA). Sera from patients with mango allergy were obtained from the hospital of Zhejiang Gongshang University. The protocol was approved by the Zhejiang Gongshang University Ethics Review Committee (2020052701), and all patients provided written informed consent. This trial was registered with the Chinese Clinical Trial Registry (ChiCTR) (www.chictr.org.cn, accessed on 29 September 2021) under ChiCTR2000034115. This trial was registered with ClinicalTrials.gov under NCT02826486. The sera used for the initial screening experiments were derived from a pool comprising equal volumes of serum from 5 individual patients with clinically confirmed mango allergies (Table S2). This pooled approach is commonly employed in exploratory epitope mapping studies to identify commonly shared IgE reactivities across a sensitized population, thereby increasing the detection signal for initial screening. Patient Selection and Diagnosis: These 5 patients were selected from the allergy clinic based on a clear clinical history of allergic reactions to mango and a positive skin prick test (SPT) to fresh mango extract (wheal diameter ≥ 3 mm compared with the negative control). SPT is a standard, validated in vivo diagnostic tool for immediate-type food allergies.

### 4.2. Instruments and Equipment

The following instruments were used in this study: an electrophoresis gel documentation and analysis system (Bio-Rad, Hercules, CA, USA); a SHZ-C water bath constant temperature shaker (Shanghai Longyue Instrument Equipment Co., Ltd., Shanghai, China); a PHS-3C pH meter (Shanghai Yidian Scientific Instrument Co., Ltd., Shanghai, China); a DYY-7C electrophoresis apparatus (LiuYi Instrument Factory, Beijing, China); and a KHB ST-360 microplate reader (Shanghai Kehua Bio-engineering Co., Ltd., Shanghai, China).

### 4.3. Methods

#### 4.3.1. BLAST Analysis of the Similarity Between Mango and Its Cross-Reactive Allergens

Potential cross-reactive proteins were predicted using the protein-protein BLAST (BLASTP) (version 2.11.0+) tool. The amino acid sequences of known mango allergens, including glyceraldehyde-3-phosphate dehydrogenase, class IV chitinase (Man i 1), profilin (Man i 4), lipoxygenase, and β–1,3-glucanase, were individually aligned against the amino acid sequences of allergenic proteins from other common foods (cow milk, egg, fish, crustaceans, mollusks, tree nuts, peanut, wheat, soybean, peach, banana, lychee, apple, pear, and carrot).

#### 4.3.2. Extraction for Mango and Other Food Proteins and Determination of Protein Concentration

Protein extracts were prepared from 14 different food sources: mango, wheat, peanut, shrimp, almond, hazelnut, pistachio, cashew, carrot, peach, lychee, pear, apple, and banana. All extractions were performed following established protocols with minor modifications. Detailed step-by-step extraction procedures for each food source, including buffers, temperatures, centrifugation conditions, and specific modifications, are provided in Supplementary Methods S1.

#### 4.3.3. Protein Quantification by Coomassie Brilliant Blue Dye-Binding Method

The protein concentration was determined using the Bradford assay [11]. Briefly, a standard curve was constructed with bovine serum albumin (BSA). The samples or standards were mixed with Coomassie Brilliant Blue G-250 reagent and incubated at room temperature for 10 min, after which the absorbance was measured at 595 nm. The protein concentration of unknown samples was calculated based on the standard curve, which showed a linear range from 1 to 20 µg (R^2^ > 0.99). All measurements were performed in triplicate.

#### 4.3.4. SDS-PAGE

A 12.5% separating gel and a 4.5% stacking gel were prepared. Samples were mixed with loading buffer at a 1:1 ratio and boiled in a water bath for approximately 10 min. A volume of 20 µL of each sample was loaded per lane. Electrophoresis was performed at a constant current of 20 mA, and the duration was controlled by monitoring the position of the tracking dye. Following electrophoresis, the gel was stained with Coomassie Brilliant Blue R-250 for 30 min and subsequently destained until protein bands were clearly visible.

#### 4.3.5. Identification of Mango Cross-Reactive Allergens by Immunoblotting of Sera from Mango-Allergic Patients

The 14 food items (mango, wheat, peanut, shrimp, almond, hazelnut, pistachio, cashew, carrot, peach, lychee, pear, apple, banana) were selected for cross-reactivity testing based on three considerations: (1) botanical relatedness to mango (Anacardiaceae: cashew and pistachio); (2) previously described clinical cross-reactivity syndromes (e.g., Rosaceae fruits in pollen-food allergy); and (3) high sequence identity (>30%) with mango allergens by BLASTp. Regardless of taxonomic distance, to systematically evaluate bioinformatic predictions. The selection of food sources for immunoblotting was primarily based on BLASTp prediction results (Table 1), prioritizing those with >30% sequence identity to mango allergens; all selected foods were also confirmed to yield measurable protein extracts by SDS-PAGE. The protein extracts from mango, wheat, peanut, shrimp, almond, hazelnut, pistachio, cashew, carrot, peach, lychee, pear, apple, and banana were subjected to SDS-PAGE separation and subsequently electrotransferred onto a polyvinylidene fluoride (PVDF) membrane. The immunoblotting procedure [21] was performed on the membrane. The transferred PVDF membrane was washed with distilled water for 5 min, followed by rinsing four times with TBST, each for 15 min at 37 °C. A blocking buffer was added, and the mixture was incubated at 37 °C for 1 h and then washed with water for 5 min. Serum diluted 1:10 from patients with mango allergy (the hospital of Zhejiang Gongshang University) was added to the plate and incubated overnight at 4 °C, followed by a 5 min water wash, each for 10 min. A 1:1000 dilution of HRP-conjugated mouse anti-human IgE secondary antibody was added, and the mixture was incubated at 37 °C for 2 h, followed by a 5 min water wash and then three washes with TBST (10 mM Tris-HCl buffer, pH 7.4, containing 0.8% NaCl and 0.1% Tween 20), each for 10 min. The PVDF membrane was immersed in a freshly prepared 4-chloro-1-naphthol solution (St. Louis, MO, USA) for color development at 37 °C for 35 min. After color development, the reaction was terminated by rinsing with high-purity water, and the membrane was air-dried between two layers of filter paper for storage. Western blotting was performed using pooled control sera from several non-allergic individuals.

#### 4.3.6. Prediction of Similar Sequences of Cross-Reactive Allergens

Based on the identification results from immunoblotting, BLAST analysis was employed to predict similar sequences with potential cross-reactive allergenic properties. The BLAST bioinformatics tool can be employed to predict potential immunological IgE-binding cross-reactivity between allergens, based on the principle of aligning the amino acid sequence of the target protein with known allergen sequences. A sequence identity > 35% to a matching counterpart is indicative of potential IgE-binding cross-reactivity. Potential cross-reactivity was screened using two thresholds: (1) the WHO/IUIS-recommended criterion of >35% identity over ≥80 amino acids; and (2) a more exploratory threshold of ≥30% identity to increase sensitivity for identifying candidate allergens, particularly those with shorter conserved regions.

#### 4.3.7. Synthesis, Purification, and Characterization of Linear Epitopes

The linear epitope peptides used in this study were synthesized using the sustainable ultrasound-assisted solid-phase peptide synthesis (SUS-SPPS) method and were N-terminally biotinylated with a 6-aminohexanoic acid spacer to enable streptavidin capture in subsequent ELISA experiments, which was recently developed and described by Mottola et al. [22,23,24]. This innovative approach integrates ultrasound irradiation into standard Fmoc-SPPS protocols, an advancement that significantly improves coupling efficiency, reduces reaction times, and minimizes solvent and reagent consumption compared with conventional methods.

Following synthesis and cleavage, the crude peptides were purified via preparative reversed-phase high-performance liquid chromatography (RP-HLC). Their identity and high purity (>95%) were confirmed by matrix-assisted laser desorption/ionization time-of-flight mass spectrometry (MALDI-TOF MS) and analytical HPLC, respectively.

With respect to the specific peptide synthesizer, the foundational SUS-SPPS methodology by Mottola et al. primarily establishes the proof-of-concept and benefits of ultrasound assistance. In our implementation, the synthesis was carried out using a Gyros Protein Technologies Symphony^®^ X (Gyros Protein Technologies, Uppsala, Sweden), which was suitably configured to accommodate the ultrasound-assisted chemistry protocol. This detail has been explicitly stated in the revised methods section.

#### 4.3.8. Characterization of Linear Epitopes of Mango Cross-Reactive Allergens by Serum IgE from Mango-Allergic Patients

The linear epitopes of mango allergens were screened for IgE reactivity. The synthesized peptides were assessed using enzyme-linked immunosorbent assay (ELISA) with serum IgE from mango-allergic patients [25]. The synthesized peptides were screened using enzyme-linked immunosorbent assay (ELISA) with serum IgE from mango-allergic patients to identify potential cross-reactive linear epitopes. The synthesized similar sequences were assayed in a 96-well microplate, each well of which was first coated with streptavidin, then sequentially received the sequences to be subjected to epitope mapping, and finally underwent antibody detection. The lyophilized peptides were dissolved in 100% dimethyl sulfoxide (DMSO); the stock solution was 10 mg/mL, and the working solution was 1 mg/mL and stored at −70 °C. The microplates were coated with 50 μL of streptavidin (5 μg/mL diluted in deionized water) per well. The plates were washed four times with PBST, and nonspecific binding sites were blocked with 0.1% BSA/PBS for 2 h at room temperature. The peptides were diluted to a final concentration of 20 μg/mL in 0.1% (*w*/*v*) BSA/PBS, and 50 μL of the peptide mixture was added to each well. The plates were incubated overnight at 4 °C. Wells without peptide solution served as controls. Primary antibody diluted in 0.1% BSA/PBS was added, and the mixture was incubated for 2 h. After four washes, horseradish peroxidase (HRP)-conjugated secondary antibody (diluted 1:800 in BSA/PBS) was added, and the samples were incubated for 2 h. The plates were subsequently washed four times, followed by the addition of 50 μL of TMB substrate per well. The reaction was terminated with 50 μL of 100 mmol/L sulfuric acid when a blue color developed. The absorbance was measured at 450 nm.

## 5. Conclusions

In summary, mango exhibits IgE-binding cross-reactivity with peanut, wheat, cashew, pistachio, and hazelnut, among which wheat and hazelnut are novel findings. Furthermore, this study innovatively identified the following cross-reactive linear epitopes: the AA80–88 sequence of mango chitinase with the AA37–45 sequence of wheat Tri a 27; the AA15–22 sequence of mango profilin with the AA65–72 sequence of pistachio Pis v 1; the AA31–51 sequence of mango profilin with the AA31–50 sequence of peanut Ara h 5; the AA50–65 sequence of mango profilin with the AA52–65 sequence of peanut Ara h 5; the AA76–96 sequence of mango profilin with the AA76–96 sequence of peanut Ara h 5; and the AA103–117 sequence of mango profilin with the AA104–117 sequence of peanut Ara h 5. Future work will focus on quantitative dose–response analyses of IgE binding to these candidate epitopes, competitive inhibition assays to confirm specificity, and ultimately, clinical correlation studies to assess their in vivo relevance.

## Figures and Tables

**Table 1:** Prediction results of mango cross-reactive allergens.

Mango Allergens	Other Allergens	Max Scores	Total Scores	Query Cover	E Value	Per Ident	AA Length
Glyceraldehyde-3-phosphate dehydrogenase	Wheat Tri a 34	602	602	98%	0.0	86.23%	337
Chitinase	Wheat Tri a 37	18.5	18.5	9%	0.042	54.55%	*240*
Chitinase	Wheat Tri a 27	20.4	20.4	17%	0.023	32.56%	*202*
Chitinase	Hazelnut Cor a 12	21.2	21.2	13%	0.009	33.33%	*159*
Chitinase	Banana Mus a 2	172	172	96%	4 × 10^−57^	41.42%	318
Profilin	Wheat Tri a 12	216	216	99%	7 × 10^−79^	75.38%	*131*
Profilin	Wheat Tri a 29	18.1	18.1	13%	0.031	33.33%	*120*
Profilin	Wheat Tri a 37	18.1	18.1	16%	0.026	40.91%	*111*
Profilin	Peanut Ara h 5	219	219	100%	2 × 10^−80^	77.1%	*131*
Profilin	Almond Pru du 4	228	228	100%	7 × 10^−84^	80.92%	*131*
Profilin	Shrimp Cra c 6	18.9	18.9	10%	0.024	50.00%	*150*
Profilin	Hazelnut Cor a 2	227	227	100%	3 × 10^−83^	80.92%	*131*
Profilin	Hazelnut Cor a 13	19.2	19.2	32%	0.013	34.09%	*140*
Profilin	Pistachio Pis v 1	18.9	18.9	14%	0.019	42.11%	*149*
Mango	Cashew	——					
Profilin	Peach Pru p 4	230	230	100%	1 × 10^−84^	81.68%	*131*
Profilin	Lychee Lit c 1	237	237	100%	2 × 10^−87^	83.97%	*131*
Profilin	Apple Mal d 4	227	227	100%	1 × 10^−83^	79.39%	*131*
Profilin	Banana Mus a 1	228	228	100%	1 × 10^−83^	81.68%	*131*
Profilin	Pear Pyr c 4	237	237	100%	2 × 10^−87^	85.50%	*131*
Profilin	Carrot Dau c 4	213	213	100%	6 × 10^−78^	75.37%	*134*
β–1,3-Glucanase	Banana Mus a 5	201	201	93%	5 × 10^−69^	57.65%	*340*

**Table 2:** Predicted sequence similarity between mango chitinase Antigenic Sequence and wheat Tri a 27.

Mango Chitinase Antigenic Sequence	Wheat Tri a 27	Score	Expect	Identities	Positives	Gaps
RDGFLNAAN (AA80–88)	RDGLLDAAN (AA37–45)	23.5 bits (48)	2 × 10^−7^	7/9 (78%)	8/9 (88%)	0/9 (0%)

**Table 3:** Predicted sequence similarity between mango Man i 4 Antigenic Sequence and peanut Ara h 5.

Mango Man i 4 Antigenic Sequence	Peanut Ara h 5	Score	Expect	Identities	Positives	Gaps
SVWAQSANFPKLNPEEITAIN (AA31–51)	SVWTESPNFPKFKPEEIAGI (AA31–50)	43.5 bits (95)	6 × 10^−12^	13/20 (65%)	14/20 (70%)	0/20 (0%)
INKDFDEPGSLAPTGL (AA50–65)	KDFEEPGHLAPTGL (AA52–65)	38.4 bits (83)	2 × 10^−10^	12/14 (86%)	13/14 (92%)	0/14 (0%)
QGEPGAVIRGKKGPGGVTVKK (AA76–96)	QGEPGVVIRGKKGTGGITIKK (AA76–96)	52.4 bits (116)	4 × 10^−15^	17/21 (81%)	19/21 (90%)	0/21 (0%)
IGIYDEPMTPGQCNM (AA103–117)	IYDEPMTPGQCNL (AA104–117)	48.6 bits (107)	3 × 10^−14^	13/14 (93%)	14/14 (100%)	0/14 (0%)

**Table 4:** Predicted sequence similarity between mango Man i 4 Antigenic Sequence and Pistachio Pis v 1.

Mango Man i 4 Antigenic Sequence	Pistachio Pis v 1	Score	Expect	Identities	Positives	Gaps
IEGHHLTA (AA15–22)	QDGHSLTA (AA65–72)	16.3 bits (31)	1 × 10^−4^	5/7 (71%)	6/7 (85%)	0/7 (0%)

## Data Availability

The original contributions presented in this study are included in the article. Further inquiries can be directed to the corresponding author.

## Supplementary Materials

The following supporting information can be downloaded at: https://www.mdpi.com/article/10.3390/ijms27114670/s1. References [26,27,28,29,30,31,32,33,34,35,36,37,38,39,40] are cited in the supplementary materials.

## Abbreviations

The following abbreviations are used in this manuscript:

FAO	Food and Agriculture Organization
BLAST	Basic Local Alignment Search Tool
BLASTP	protein-protein BLAST
CAC	Codex Alimentarius Commission
AA	amino acid
SDS-PAGE	sodium dodecyl sulfate-polyacrylamide gel electrophoresis
PVDF	polyvinylidene fluoride
TBST	Tris-buffered saline with Tween
HRP	horseradish peroxidase
TMB	3,3′,5,5′-tetramethylbenzidine
ELISA	enzyme-linked immunosorbent assay
DMSO	dimethyl sulfoxide
BSA	bovine serum albumin
PBS	phosphate-buffered saline
PBST	PBS with Tween-20
IgE	immunoglobulin E
