# Supplementary material for: Identification of Mango Cross-Reactive Allergens and Cross-Reactive Linear Epitopes Using Serum from Patients with Mango Allergy
Source: Int J Mol Sci. 2026 May 22;27(11):4670. doi: 10.3390/ijms27114670 (PMC13256811; doi:10.3390/ijms27114670)
Supplement: Supplementary file 1 [file ijms-27-04670-s001.zip › ijms-4302168-supplementary.pdf]

Supplementary Materials

Supplementary Methods S1: Detailed Protein Extraction Protocols

This supplement provides the complete extraction procedures for all food protein samples used in this study. The corresponding protein concentration results are presented in the main text (Section 3.1, Table 1).

2.3.2. Extraction for Mango and Other Food Proteins and Determination of Protein Concentration

2.3.2.1. Extraction of Mango Proteins

Fresh, commercially ripe mango fruits of uniform size and free from visual defects or rot were peeled, and the pulp was homogenized using a blender to create a homogenate for subsequent use. The method for extracting mango proteins was slightly modified from the protocols described by Dube et al. [1] and Hou et al. [2]. Briefly, the homogenate was mixed with acetone at a 1:3 ratio (w/v) and stirred for 2 h. The mixture was then centrifuged at 8000 rpm and 4 °C for 20 min. After the acetone was evaporated from the precipitate, 2 g of the resulting powder was stirred with 30 mL of 0.02 M phosphate buffer (pH 7.4) containing 0.13 M sodium chloride for 6 h for extraction at 4 °C. The suspension was subsequently centrifuged at 8000 rpm and 4 °C for 10 min. The supernatant was collected and dialyzed overnight against 10 mM sodium phosphate buffer (pH 7.4). The final protein extract was aliquoted and stored. The protein concentration was determined at 595 nm using the Coomassie Brilliant Blue assay.

2.3.2.2. Extraction of Wheat Proteins

Wheat grains of uniform size and free from visible defects, pests, and molds were selected. The grains were milled into whole wheat flour, which was then defatted prior to extraction. Wheat protein was extracted according to the methods of Zhao et al. [3] with minor modifications. Briefly, wheat

grains were ground into powder and defatted with acetone at a ratio of 1:10 (w/v) for 6 h. Defatted wheat flour was added to a 40% ethanol solution of sodium carbonate–sodium bicarbonate buffer (20 mM, pH 9.6; 0.5 M NaCl; 1 mM phenylmethylsulfonyl fluoride [PMSF]) at a ratio of 1:10 (m/v) and stirred at room temperature for extraction for 10 h. The resulting slurry was centrifuged at 4 °C and 9,000 rpm for 30 min. The supernatant was collected, and the protein concentration was determined using the Coomassie Brilliant Blue method, with measurements taken at 595 nm.

2.3.2.3. Extraction of Peanut Proteins

Sound and mature peanut kernels of uniform size and free from mold, insect damage, or mechanical injury were selected. In accordance with the extraction method of Zhang [4], after the seed coat was removed, the kernels were ground into powder using a milling machine. The powder was added to acetone at a ratio of 1:10 (m/v), shaken for defatting at room temperature for 6 h, and then centrifuged for 10 min (12,000 ×g). After acetone was removed from the precipitate, 20 mM phosphate buffer (containing 1 M NaCl, pH 7.8) was added at a ratio of 1:20 (m/v), followed by shaking at room temperature for extraction for 4 h. The mixture was then centrifuged (4 °C, 14,000 × *g*, 10 min). The supernatant was dialyzed against ultrapure water at 4 °C for 4 h and then stored frozen for later use. The protein concentration was determined using the Coomassie Brilliant Blue method at 595 nm.

2.3.2.4. Extraction of Shrimp Proteins

Fresh Antarctic krill samples that were intact and free from discoloration or odor were selected. In accordance with the methods of Yin et al. [5], 4 g of krill meat was weighed, and deionized water was added at a ratio of 3.15:1 (m/v). The pH was subsequently adjusted to 11.38 using the addition of 2 mol/L sodium hydroxide, and the mixture was left to stand for 1.5 h. The mixture was then centrifuged at 10,000 ×g for 10 min at 4 °C to obtain the supernatant. The entire extraction process was repeated three times. Finally, the collected supernatant was adjusted to pH 4.5 with 2 mol/L phosphoric acid, left to stand for 1.5 h, and centrifuged at 10,000 ×g for 10 min at 4 °C to collect the precipitate. The protein concentration was determined using the Coomassie Brilliant Blue method at 595 nm.

2.3.2.5. Extraction of Almond Proteins

Sound, dry almond kernels of uniform size and free from mold, insect damage, or rancidity were selected. In accordance with the methods in reference [6], raw almonds were ground into a fine powder and then defatted by shaking with n-hexane at a ratio of 1:5 (w/v) for 3 h. After n-hexane

was removed from the defatted powder, the mixture was shaken with carbonate buffer (0.05 mol/L, pH 9.6) at a ratio of 1:250 (w/v) for extraction for 1 h at 50 °C. The crude extract was then centrifuged at 16,000 ×g for 30 min at 4 °C. The supernatant was collected for subsequent use. The protein concentration was determined using the Coomassie Brilliant Blue method at 595 nm. All the determinations were repeated three times, and the average value was calculated.

2.3.2.6. Extraction of Hazelnut Proteins

Sound hazelnut kernels of uniform size and free from mold, insect damage, or rancid odor were selected and ground into powder. In accordance with the extraction method of Fang et al. [7], 30 g of hazelnut powder was mixed with n-hexane at a ratio of 1:15 (w/v) for defatting. The mixture was shaken for extraction for 6 h and then centrifuged twice at 8000 r/min for 10 min. The supernatant was removed to obtain the precipitate. After removing the n-hexane from the precipitate, ultrapure water was added at a ratio of 1:6 (w/v). The mixture was homogenized at high speed (12,000 r/min) for 2 min using a magnetic stirrer. After homogenization, the pH was adjusted to 8.0 with dilute alkali (1% NaOH), followed by water bath extraction at 40 °C with shaking for 4 h. The mixture was then centrifuged (6000 r/min, 10 min) to separate the phases, and the supernatant was collected for subsequent use. The protein concentration was determined using the Coomassie Brilliant Blue method at 595 nm.

2.3.2.7. Extraction of Pistachio Proteins

Sound pistachio kernels of uniform size and free from mold, insect damage, or rancidity were selected and ground into powder. In accordance with the extraction method of Noorbakhsh et al. [8], 10 g of pistachio powder was mixed with n-hexane at a ratio of 1:15 (w/v) for defatting. The mixture was shaken for extraction for 6 h and then centrifuged (8000 r/min for 20 min). The supernatant was removed to obtain the precipitate. After n-hexane was removed from the precipitate, it was mixed with phosphate buffer (20 mmol/L, pH 7.4) at a ratio of 1:10 (w/v). The mixture was stirred uniformly and shaken at 4 °C for extraction for 6 h and then centrifuged at 4 °C and 9000 ×g for 30 min. The supernatant was collected for subsequent use. The protein concentration was determined using the Coomassie Brilliant Blue method at 595 nm.

2.3.2.8. Extraction of Cashew Proteins

In accordance with the extraction method of Zhong et al. [9], plump cashew nuts that were uniform in texture and free from disease and insect

damage were selected prior to the experiment. A certain amount of cashew nuts was crushed in a grinder for subsequent use. The powder was defatted by shaking with n-hexane at a ratio of 1:15 (w/v) for 6 h, followed by centrifugation (8000 r/min; 20 min). The supernatant was removed to obtain the precipitate. After removing the n-hexane from the precipitate, 1 g was removed, and 40 mL of distilled water was added. The pH was adjusted to 9 with a 0.01 mol/L NaOH solution, and the mixture was stirred until it dissolved. Extraction was performed at 35 °C for 1.5 h, followed by centrifugation at 4000 r/min for 15 min. The supernatant was collected for protein content determination.

2.3.2.9. Extraction of Carrot Proteins

In accordance with the extraction method of Guo [10], vigorously growing carrots of uniform size that were free from mechanical damage, pests, and disease were selected, washed, peeled, and homogenized using a blender. The homogenate was mixed with acetone at a ratio of 1:4 (w/v) and magnetically stirred for 3 h, followed by centrifugation at 12,000 r/min for 10 min at 4 °C. The supernatant was discarded. After acetone was removed from the precipitate, phosphate buffer (0.02 mol/L, pH 7.2) was added at a ratio of 1:10 (w/v) for extraction for 2 h (at 4 °C). The mixture was then centrifuged at 4 °C and 15,000 r/min for 20 min, and the supernatant was collected for subsequent use. The protein concentration was determined using the Coomassie Brilliant Blue method at 595 nm.

2.3.2.10. Extraction of Peach Proteins

In accordance with the extraction method of Pasini et al. [11], ripe peach fruits of uniform size that were free from blemishes or rot were selected. The pulp was collected and homogenized, and the resulting homogenate was mixed with acetone at a ratio of 1:3 (w/v) for defatting for 5 h. The defatted sample was then dissolved in 0.01 mol/L phosphate buffer (0.015 mol/L sodium chloride, pH 7.2) at a ratio of 1:10 (w/v), followed by shaking extraction for 2 h (at 40 °C). The mixture was centrifuged (10,000 ×g, 30 min, 4 °C), and the supernatant was collected and stored at −20 °C for subsequent use. The protein concentration was determined using the Coomassie Brilliant Blue method at 595 nm.

2.3.2.11. Extraction of Lychee Proteins

Fresh lychee fruits at commercial maturity with intact pericarp (bright red) and uniform size that were free from cracking or browning were selected.

In accordance with the extraction method of Song et al. [12], the pulp was collected and homogenized, and 3 g of the homogenate was mixed with 15 mL of cold acetone and subjected to shaking extraction for 2 h, followed by centrifugation at 15,000 × g for 10 min at 4 °C. This step was repeated twice. After acetone was removed from the precipitate, it was mixed with phosphate buffer (0.01 M, pH 7.4, 0.15 M NaCl) at a ratio of 1:15 (w/v) and extracted with shaking at 4 °C. After centrifugation (20,000 × g, 45 min), the supernatant was collected and stored frozen for subsequent use. The protein concentration was determined using the Coomassie Brilliant Blue method at 595 nm.

2.3.2.12. Extraction of Pear Proteins

Fresh fragrant pear fruits with intact skin, uniform size, and no bruising or rot at commercial maturity were selected. In accordance with the extraction method of Zhang et al. [13], the pulp was collected and homogenized, and the fresh homogenate was mixed with solvent at a solid-to-liquid ratio of 1:10 (w/v). The pH was adjusted to 10 with 0.1 mol/L NaOH. Extraction was performed at 40 °C with constant-temperature shaking in a water bath for 2 h. The mixture was centrifuged at 8000 r/min for 15 min, and the supernatant was collected for subsequent use. The protein concentration was determined using the Coomassie Brilliant Blue method at 595 nm.

2.3.2.13. Extraction of Apple Proteins

Fresh apple fruits of intact skin, uniform size, and free from bruising or rot at commercial maturity were selected. In accordance with the extraction method of Deng et al. [14], the pulp was collected and homogenized, and 1 g of the homogenate was mixed with 25 mL of 100 mmol/L Tris-HCl buffer

(pH 9.0) and magnetically stirred for 2 h (at 4 °C). The mixture was then centrifuged at 4 °C and 12,000 × g for 20 min. The supernatant was collected and stored at −20 °C for subsequent use. The protein concentration was determined using the Coomassie Brilliant Blue method at 595 nm.

2.3.2.14. Extraction of Banana Proteins

Fresh banana fruits at the fully ripe but firm stage (peel yellow color with minimal brown spots), with intact peels and a uniform size, were selected. In accordance with the methods of Nikolic et al. [15], the fruits were peeled, and 160 g of the pulp was homogenized in 300 mL of 50 mM NH_4HCO_3 buffer (pH 8.5) and extracted for 2 h. Clarification of the protein extract was achieved by centrifugation at 3000 × g for 15 min at 4 °C. The supernatant was collected, dialyzed against ammonium bicarbonate buffer (50 mM NH_4HCO_3, pH 8.5) for 48 h, aliquoted, and stored frozen for subsequent use. The protein concentration was determined using the Coomassie Brilliant Blue method at 595 nm.

Supplementary Figure S1

Figure S1. Standard curve for protein concentration determination by the Bradford method. Bovine serum albumin (BSA) was used as the standard. The linear regression equation and R^2 value are shown.

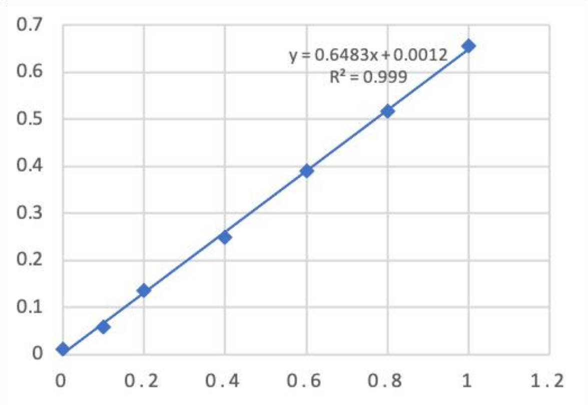

Figure S1. Standard curves of mango and cross-allergen proteins.

Note: The abscissa represents the protein concentration (mg/mL), and the ordinate represents the OD value.

Table S1. Protein concentration results of mango and mango cross-allergen protein.

	**OD595 nm**	**Dilution factor**	**Protein Concentrati (mg/mL)**
**Mango**	0.253 ±0.004	——	0.389 ±0.004
**Wheat**	0.098 ±0.018	1:10	1.495 ±0.129
**Peanut**	0.255 ±0.033	1:45	17.607 ±1.017
**Shrimp**	0.245 ±0.012	1:40	15.059 ±0.359
**Almond**	0.200 ±0.006	1:40	12.247 ±0.204
**Hazelnut**	0.486 ±0.008	1:50	37.372 ±0.319
**Pistachio**	0.342 ±0.033	1:15	7.875 ±0.339

**Cashew**	0.358 ±0.009	1:10	5.498 ±0.070
**Carrot**	0.038 ±0.007	——	0.056 ±0.006
**Peach**	0.043 ±0.002	——	0.065 ±0.002
**Lychee**	0.118 ±0.009	——	0.180 ±0.007
**Crystal Pear**	0.270 ±0.008	——	0.415 ±0.006
**Fragrant Pear**	0.164 ±0.009	——	0.251 ±0.007
**Apple**	0.048 ±0.017	——	0.072 ±0.012
**Banana**	0.058 ±0.008	——	0.088 ±0.006

Table S2 Clinical characteristics of the five mango-allergic patients whose sera were pooled for this study.

Patient ID	Age (years)	Sex	Primary mango allergy symptoms	Severity grade	SPT wheal diameter (mm)	Mango-specific IgE (kU_A/L)	Known clinical cross-reactions to tested foods*
**P1**	**23**	**F**	Oral allergy syndrome (itching of lips and mouth)	Mild	**5**	**12.3**	None
**P2**	**21**	**F**	Urticaria (generalized hives)	Moderate	**8**	**25.57**	None
**P3**	**23**	**M**	Gastrointestinal symptoms (nausea, abdominal pain)	Mild	**4**	**5.8**	None

**P4**	**20**	**F**	Orofacial swelling and rhinitis	Modera te	**6**	**18.4**	None (but reported mild OAS to kiwi, not tested in this study)
**P5**	**21**	**M**	Oral allergy syndrome + urticaria	Mild	**7**	**9.2**	None

Footnotes:

SPT = skin prick test using fresh mango extract, wheal diameter measured 15 min after test; positive defined as ≥3 mm larger than negative control.

Mango-specific IgE levels were quantified by ImmunoCAP (Thermo Fisher Scientific); values >0.35 kU_A/L are considered positive.

Severity grade was assessed by the attending allergist based on clinical presentation: Mild = symptoms limited to oropharynx or skin; Moderate = skin or gastrointestinal symptoms without respiratory distress or hypotension.

*Tested foods in this study include wheat, peanut, cashew, pistachio, hazelnut, almond, carrot, peach, lychee, pear, apple, banana, and shrimp. None of the five patients reported prior clinical reactions to any of these foods based on detailed medical history.

References

1. Dube, M.; Zunker, K.; Neidhart, S.; Carle, R.; Steinhart, H.; Paschke, A. Effect of technological processing on the allergenicity of mangoes (Mangifera indica L.). *J. Agric. Food Chem.* **2004**, *52*, 3938–3945.

2. Hou, L.Y. Mango allergen components analysis by three different extracting methods. *Chin. J. Food Hyg.* **2014**, 417–421.

3. Zhao, J.; Li, Z.; Khan, M.U.; Gao, X.; Yu, M.; Gao, H.; Li, Y.; Zhang, H.; Dasanayaka, B.P.; Lin, H. Extraction of total wheat (Triticum aestivum) protein fractions and cross-reactivity of wheat allergens with other cereals. *Food Chem.* **2021**, *347*, 129064.

4. Zhang, Y. Evaluation of changes in protein allergenicity in fresh peanuts during thermal processing. Master's thesis, Nanchang University, Nanchang, China, 2019.

5. Yin, L.a.; Jiang, X.; Fan, Y.; Wang, J.; Xue, C.; Xue, Y. Preparation, Gel electrophoresis analysis, and nutritional evaluation of a functional krill protein concentrate with low fluoride level from Antarctic krill (Euphausia superba). *J. Aquat. Food Prod. Technol.* **2017**, *26*, 958–968.

6. L'Hocine, L.; Pitre, M. Quantitative and qualitative optimization of allergen extraction from peanut and selected tree nuts. Part 1. Screening of optimal extraction conditions using a D-optimal experimental design. *Food Chem.* **2016**, *194*, 780–786.

7. Fang, Y.; Li, J.; Zhu, X.; Li, X.; Xu, S.; Wu, H.; Zhang, H.; Luo, Y. Optimization and purification of natural protein extract from hazelnut press cake and its antioxidant activity. *Front. Nutr.* **2025**, *12*, 1636534.

8. Noorbakhsh, R.; Mortazavi, S.A.; Sankian, M.; Shahidi, F.; Maleki, S.J.; Nasiraii, L.R.; Falak, R.; Sima, H.R.; Varasteh, A. Influence of processing on the allergenic properties of pistachio nut assessed in vitro. *J. Agric. Food Chem.* **2010**, *58*, 10231–10235.

9. Zhong, J.Z.; Dun, R.Y.; Huang, Z.L.; Liu, W.; Liu, C.M. Optimizing the extraction of protein from cashew nuts. *J. Food Sci.* **2014**, *35*, 18–22.
10. Guo, Z.B. Somatic embryogenesis and protein expression in maize and carrot. *J. Anhui Agric. Sci.* **2010**, *38*, 9979–9980.
11. Pasini, G.; Curioni, A.; Vegro, M.; Pagani, M.; Masi, A.; Schievano, E.; Antico, A. Extraction and mass spectrometry identification of a major peach allergen Pru p 1. *J. Sci. Food Agric.* **2012**, *92*, 570–576.
12. Song, J.J.; Zhang, H.Y.; Liu, Z.G.; Ran, P.X. Cloning of the panallergen profilin from lychee fruit and its cross-reactivity with birch pollen profilin Bet v 2. *Food Agric. Immunol.* **2007**, *18*, 129–138.
13. Zhang, Q.; Li, L.; Li, Y.T.; Wang, X.J.; Hailiqian, T.E.D.H.; Yang, F. Study on the optimum extraction technology of protein from korla pear. *J. Food Saf. Qual.* **2020**, *11*, 4643–4648.
14. Deng, L.L.; Pan, X.Q.; Sheng, J.P.; Shen, L. Optimization of conditions for determining micro-soluble protein content in apple tissue by Coomassie brilliant blue method. *J. Food Sci.* **2012**, *33*, 185–189.
15. Nikolić, J.; Nešić, A.; Kull, S.; Schocker, F.; Jappe, U.; Gavrović-Jankulović, M. Employment of proteomic and immunological based methods for the identification of catalase as novel allergen from banana. *J. Proteomics* **2018**, *175*, 87–94.
